# Genome-Wide Association Study of Age at First Calving in U.S. Holstein Cows

**DOI:** 10.3390/ijms24087109

**Published:** 2023-04-12

**Authors:** Dzianis Prakapenka, Zuoxiang Liang, Yang Da

**Affiliations:** Department of Animal Science, University of Minnesota, Saint Paul, MN 55108, USA

**Keywords:** age at first calving, GWAS, SNP, additive effect, dominance effect, reproductive hormone

## Abstract

A genome-wide association study (GWAS) of age at first calving (AFC) using 813,114 first lactation Holstein cows and 75,524 SNPs identified 2063 additive effects and 29 dominance effects with *p*-values < 10^−8^. Three chromosomes had highly significant additive effects in the regions of 7.86–8.12 Mb of Chr15, 27.07–27.48 Mb and 31.25–32.11 Mb of Chr19, and 26.92–32.60 Mb of Chr23. Two of the genes in those regions were reproductive hormone genes with known biological functions that should be relevant to AFC, the sex hormone binding globulin (*SHBG*) gene, and the progesterone receptor (*PGR*) gene. The most significant dominance effects were near or in *EIF4B* and *AAAS* of Chr05 and *AFF1* and *KLHL8* of Chr06. All dominance effects were positive overdominance effects where the heterozygous genotype had an advantage, and the homozygous recessive genotype of each SNP had a very negative dominance value. Results from this study provided new evidence and understanding about the genetic variants and genome regions affecting AFC in U.S. Holstein cows.

## 1. Introduction

Age at first calving (AFC) is measured in negative days, such that a larger AFC value represents a younger first-calving age and a smaller AFC value represents an older first-calving age. This is a new reproduction trait for U.S. Holstein genomic evaluation [1,2]. A large sample of Holstein cows with AFC phenotypic values and genotypic data of single nucleotide polymorphism (SNP) markers has become available, providing an opportunity to identify genetic variants and genome regions that affect AFC and involve puberty and successful pregnancy with high statistical confidence. Prior to the inclusion of AFC for genomic evaluation, the U.S. Holstein genomic evaluation included three reproductive traits, daughter pregnancy rate (DPR), which is the percentage of cows that become pregnant during each 21-d period, and cow conception rate (CCR) and heifer conception rate (HCR), each as percentage pregnancy at each service [3]. These reproductive traits should involve different and possibly some overlapping physiological processes affecting reproduction, and a genome-wide association study (GWAS) using large samples is a powerful approach to identify and understand the genetic factors underlying these reproductive traits. We previously reported results from a large-scale GWAS for DPR, CCR, and HCR [4], but a similar large-scale GWAS was unavailable for the AFC of U.S. Holstein cows. The sample size for AFC is now much larger than those for the large-scale GWAS for DPR, CCR, and HCR. The purpose of this study was to identify genetic variants and chromosome regions affecting AFC in U.S. Holstein cows using a large sample from the U.S. Holstein genomic evaluation data.

## 2. Results and Discussion

The results presented below focus on the three chromosome regions with the most significant additive effects and the two chromosome regions with the most significant dominance effects. The top 100 additive effects are provided in Table S2, and all significant dominance effects are provided in Table S3.

### 2.1. Additive Effects

**Figure 1:**
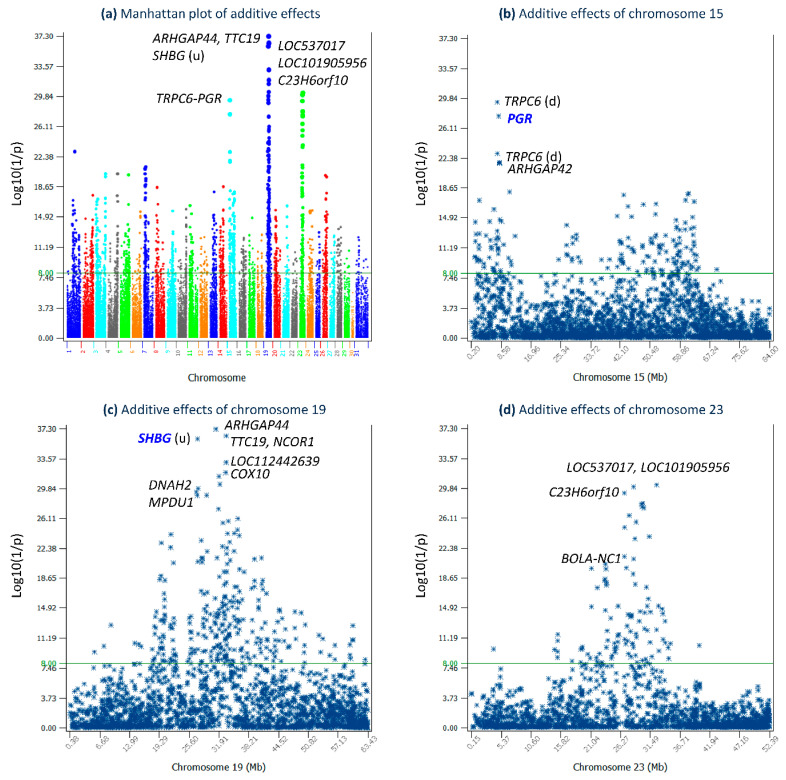
Graphical view of additive effects. (**a**) Manhattan plot of additive effects of all chromosomes. (**b**) Additive effects of chromosome 15. (**c**) Additive effects of chromosome 19. (**d**) Additive effects of chromosome 23. ‘u’ indicates the SNP is upstream of the gene, and ‘d’ indicates the SNP is downstream of the gene.

The GWAS analysis identified 2063 additive effects with log_10_(1/*p*) > 8. The observed log_10_(1/*p*) values of all SNPs were shown in the Manhattan plot of Figure 1a. Highly significant additive effects involved three chromosomes, the 7.86–8.12 Mb region of Chr15, 27.07–27.48 Mb and 31.25–32.11 Mb regions of Chr19, and 26.92–32.60 Mb region of Chr23 (Figure 1b–d, Table 1). Two of the genes in those regions were sex hormone genes with known biological functions that should be relevant to AFC, the sex hormone binding globulin (*SHBG*) gene, and the progesterone receptor (*PGR*) gene. The known biological functions and the statistical significance of the SNPs in or near these two genes implicated the involvement of the two reproductive hormone genes in the AFC of Holstein cows.

The 7.86–8.12 Mb region of Chr15 had three genes, *TRPC6*, *PGR*, and *ARHGAP42*. Of these three genes, *PGR*, as the progesterone receptor gene, has known relevant biological functions affecting AFC. This gene encodes a member of the steroid receptor superfamily, and the encoded protein mediates the physiological effects of progesterone, which plays a central role in reproductive events associated with the establishment and maintenance of pregnancy [5]. *PGR* had four SNPs, and one of these SNPs (rs136764006) had log_10_(1/*p*) = 27.63 (Table 1). *TRPC6* had four SNPs, and none of those four SNPs reached the statistical significance of log_10_(1/*p*) > 8, but an SNP about 3151 bp downstream of *TRPC6* or 349,081 bp upstream of *PGR* was highly significant with log_10_(1/*p*) = 29.38 (Figure 1b, Table 1). *ARHGAP42* had 14 SNPs, and four of the 14 SNPs about 200 Kb downstream of *PGR* were significant with log_10_(1/*p*) values of 21.74–21.89 (Figure 1b). The *TRPC6* and *ARHGAP42* genes did not have known biological functions directly affecting AFC.

The 27.07–27.48 Mb region of Chr19 had three genes, *MPDU1*, *SHBG*, and *DNAH2*. In this region, the most significant SNP was *rs111004845*, which was 9664 bp upstream of *SHBG*, noting that *SHBG* did not have any SNP in our dataset. *SHBG* is the sex hormone–binding globulin gene and the only gene in this region known to have a biological function related to reproduction. This gene encodes a steroid-binding protein, and the encoded protein transports androgens and estrogens in the blood, binding each steroid molecule as a dimer formed from identical or nearly identical monomers [6]; the sex hormone binding globulin was likely associated with early puberty [7,8]. The known biological function of *SHBG* affecting reproduction and the highly significant SNP in the proximity of *SHBG* should implicate *SHBG* as an interesting candidate gene affecting AFC. *MPDU1* had one SNP, and *DNAH2* had four SNPs in our dataset.

The 31.25–32.11 Mb regions of Chr19 had the most significant additive effect in *ARHGAP44* with log_10_(1/*p*) = 37.30 (Table 1), but *ARHGAP44* was not known to affect reproduction. The *HS3ST3A1* gene is widely expressed, with the most abundant expression in the liver and placenta [9], and the gene expression in the placenta could affect AFC. This gene had an additive effect with log_10_(1/*p*) = 27.34 (Table S1). In this region, *TTC19*, *NCOR1*, and *COX10* had highly significant additive effects.

In the 26.92–32.60 Mb of Chr23, the most significant SNPs were in three genes with unknown functions, *LOC537017*, *LOC101905956*, and *C23H6orf10* (Table 1). This relatively large chromosome region (6.32 Mb in size) had multiple genes with or near highly significant SNP effects (log_10_(1/*p*) > 20), but only the non-classical MHC class I gene of *BOLA-NC1* was reported to affect reproduction [10,11,12,13].The significant SNP closest to *BOLA-NC1* was *rs110855962* with log_10_(1/*p*) = 21.02 (Figure 1d).

Among the top 20 SNPs, the sizes of positive allelic effects were in the range of 0.354–0.818, the sizes of the negative allelic effects were −0.363 to −0.270, and the absolute values of the additive effects (α) were in the range of 0.98–1.04 days (Table 1). Such effect sizes were considerably smaller than some of the dominance effects described below.

### 2.2. Dominance Effects

**Figure 2:**
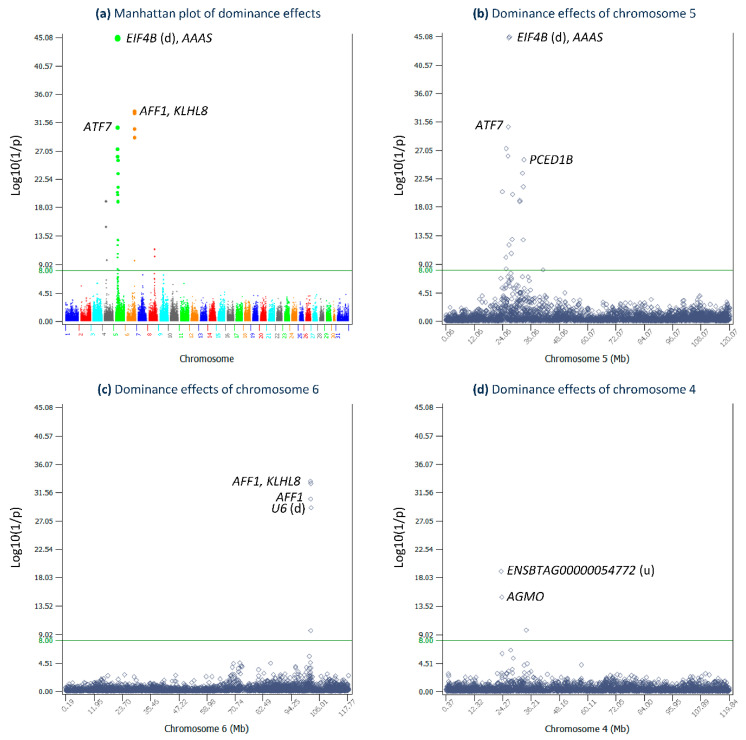
Graphical view of dominance effects. (**a**) Manhattan plot of dominance effects of all chromosomes. (**b**) Dominance effects of chromosome 5. (**c**) Dominance effects of chromosome 6. (**d**) Dominance effects of chromosome 4. ‘u’ indicates the SNP is upstream of the gene, and ‘d’ indicates the SNP is downstream of the gene.

The GWAS analysis identified 29 dominance effects with log_10_(1/*p*) > 8. The observed log_10_(1/*p*) values of all SNPs are shown in the Manhattan plot of Figure 2a. The most significant dominance effects were located in the 26.38–26.96 Mb region of Chr05 and the 101.86–102.17 Mb region of Chr06 (Figure 2a–c).

The 26.38–26.96 Mb region of Chr05 had the most significant dominance effect of 14,636 bp downstream of *EIF4B* (Figure 2b, Table 2), noting that *EIF4B* did not have any SNP in our dataset, and this dominance effect with log_10_(1/*p*) = 45.08 was the most significant effect among all additive and dominance effects. The second-most significant dominance effect was that in *AAAS* on Chr05 (Table 2), and this effect also was the second-most significant effect among all additive and dominance effects. We previously showed the SNPs in this 26.38–26.96 Mb region of Chr05 had significant dominance effects for milk, fat, and protein yields [4].

The 101.86–102.17 Mb region of Chr06 had three highly significant dominance effects (log_10_(1/*p*) > 30) in *AFF1* and *KLHL8* (Figure 2c, Table 2). *AFF1* had eight SNPs in our dataset, and two of these SNPs had log_10_(1/*p*) > 30. One of the two significant SNPs (rs43480825) in *AFF1* present in a previous Holstein GWAS was the most significant dominance effect for heifer conception rate (HCR) and the second-most significant dominance effect for daughter pregnancy rate (DPR) and cow conception rate (CCR) [4]. *KLHL8* had seven SNPs, and one of these SNPs had log_10_(1/*p*) > 30. The *KLHL8* gene was proposed as a candidate gene for nonreturn rate in Holstein heifers [14]. The dominance effects in *AFF1* and *KLHL8* were positive overdominance effects and had the same pattern as the positive overdominance effects near *EIF4B* and in *AAAS* of *Chr05*.

These positive overdominance effects of AFC had five features. First, each SNP had a ‘recessive allele’ that, in homozygous status, had a very negative dominance value. Second, each SNP had a ‘dominant allele’ that, in heterozygous status, neutralized the negative effect of the recessive allele. Third, the dominant allele in homozygous status behaved like a neutral allele with a small absolute dominance value or dominance deviation, noting that each dominance value was a deviation of the genotypic value from the mean and additive value. Fourth, the dominance value of the heterozygous genotype was more positive than that of either homozygous genotype, but the heterozygous dominance value was not much above zero. Fifth, the recessive allele had an allele frequency of mostly <0.10, so the homozygous recessive genotype was rare and had a genotypic frequency of mostly <0.01 (Table 2). For the example of *rs109438971*, which had the most significant dominance effect, the dominance value of the homozygous genotype of the recessive allele (allele 1) was −9.76, compared to the slightly negative dominance value −0.06 of the homozygous genotype of the dominant allele (allele 2) and the positive dominance value 0.62 of the heterozygous genotype with alleles 1 and 2. This positive value was less than 1/15 of the negative recessive homozygous genotypic value, but the heterozygous genotypic frequency was about 30 times that of the homozygous recessive genotype (0.152 vs. 0.005). Consequently, at the population level, the heterozygous advantage in the form of a positive overdominance effect more than offset the very negative effect of the homozygous recessive genotype. The contribution to the population mean of the *rs109438971* dominance values was (d_DR)(f_DR) = (0.622)(0.152) = 0.095 for the heterozygous genotypes and (d_RR)(f_RR) = (−9.76)(0.005) = −0.049 for the homozygous recessive genotypes, based on the dominance values and genotypic frequencies in Table 2. Therefore, the positive contribution of the heterozygous genotypes to the population mean of the *rs109438971* dominance values was about twice the negative contribution of the homozygous recessive genotypes. This heterozygous advantage in the form of a positive overdominance effect likely was the reason the very negative recessive allele still had a substantial allele frequency of 0.081 (Table 1) and was not eliminated over the years.

Compared to the additive effects in Table 1, the recessive genotypes were considerably more detrimental than negative additive effects. For the top dominance effects, the dominance values of the recessive genotypes were in the range of −9.76 to −5.18 (Table 2), whereas the allelic effects of the negative alleles of additive effects were in the range of −0.363 to −0.270 for the top 20 additive effects (Table 1). Given that the negative dominance values of the recessive genotypes were more than ten times as large as the negative allelic effects, the first step of the application of the GWAS results would be the use of the recessive SNP genotypes for heifer culling.

### 2.3. Elimination of Rare Negative Recessive Genotypes for Heifer Culling

The results of the dominance effects of AFC identified seven SNPs with very negative dominance values for the recessive homozygous genotypes (Table 2). We recommend using the recessive SNP genotypes of these seven SNPs for culling heifers that carry such genotypes. Detailed results supporting this recommendation are provided in Table S4. Among the 813,114 cows in this study, 3541–5274 cows carried the negative recessive genotypes for at least one of the seven SNPs for heifer culling (Table S4). For dominance values that removed additive values, the heterozygous genotypes had the highest dominance values (Table 2). To evaluate the impact of culling heifers with the recessive genotypes, we defined the negative impact of a recessive genotype as the difference between the average of the phenotypic values of cows carrying the recessive genotype and the average of the phenotypic values of cows carrying the heterozygous genotype and the homozygous dominant genotype of each SNP. The results of negative impact showed that cows with the recessive genotypes required 7.69–12.83 days longer than the heterozygous genotypes and homozygous dominant genotypes for first calving and had sharply lower yields, 201.23–646.33 kg lower for milk yield, 9.05–26.03 kg lower for fat yield, and 6.74–19.27 kg lower for protein yield (Table 3). Therefore, evidence from this study showed that the recessive genotypes had severely negative effects on AFC and the yield traits and that heifers with the recessive genotypes should be culled. We are not ready to recommend the elimination of bulls carrying the recessive alleles because such a recommendation requires a separate study.

### 2.4. Comparison with Previous Studies

Several GWASs on AFC were available prior to our study, but results from the previous studies, including a study in beef cattle [15] and a study in Chinese Holsteins [16], did not overlap the results from our study. The beef study using 185,356 Nellore heifers identified significant SNPs on chromosomes 2 and 14, and none of those significant SNPs was highly significant in our Holstein study. Results from the Chinese Holsteins using 19,111 heifers also lacked overlap with the results of our study. Although the exact reasons for the differences among those studies were unknown, results from our study add new understanding about the genetic variants and chromosome regions underlying AFC from a large sample of U.S. Holstein cows. In comparison with our previous GWAS results for three other reproductive traits (DPR, CCR, and HCR) in U.S. Holstein cows [4], AFC did not share significant additive effects and only shared a significant dominance effect of *rs43480825* in *AFF1* with DPR, CCR, and HCR. This limited sharing of common significant effects indicated that AFC mostly involved different genetic mechanisms from those for DPR, CCR, and HCR.

### 2.5. Gene Ontology of Candidate Genes

To understand the potential biological functions of the candidate genes, we searched Gene Ontology Resources [17], KEGG [18] and DAVID [19] for the biological processes involved by the 14 candidate genes for additive effects in Table 1 and nine candidate genes for dominance effects in Table 2. However, Gene Ontology Resources had more details than available from KEGG and DAVID. Therefore, we only included the biological processes involved by the candidate genes from Gene Ontology Resources in Table S5 for candidate genes of additive effects with 560 entries and in Table S6 for candidate genes of dominance effects with 486 entries. Other than *SHBG*, for which no descriptions of its biological functions were available other than the hormone binding process indicated by the gene name, every candidate gene was involved in multiple biological processes. Although any of those processes could have affected AFC, the exact genetic mechanisms of the significant SNP effects remained unknown. Among all the biological processes, only *PGR* and *AAAS* were involved in known reproductive processes. The *PGR* gene was already known for its role in the pregnancy process, which should be highly relevant to AFC, and was one of the multiple reproductive processes described for *PGR* in Table S5. The *AAAS* gene was involved in fertilization and the reproductive process (Table S6), which should also be highly relevant to AFC.

## 3. Materials and Methods

### 3.1. Holstein Population and SNP Data

The Holstein population in this study had 813,114 first lactation cows with AFC phenotypic observations and 78,964 original and imputed SNPs. With the requirement of 0.05 minor allele frequency, 75,524 SNPs were used in the GWAS analysis. The SNP positions were those from the ARS-UCD1.2 cattle genome assembly. Genes containing or in the proximity of highly significant additive and dominance effects were identified as candidate genes affecting AFC. The AFC phenotypic values are reported in negative days, such that higher AFC values represent younger first-calving ages and are considered more desirable than lower AFC values, representing older first-calving ages [1,2]. The AFC phenotypic values used in the GWAS analysis were the phenotypic residuals after removing fixed nongenetic effects available from the December 2021 U.S. Holstein genomic evaluation data. The 813,114 phenotypic residuals values had an approximate bell-shaped distribution (Figure S1; Supplementary Materials), and the basic statistics of these phenotypic values are described in Table S1.

### 3.2. GWAS Analysis

The GWAS analysis used an approximate generalized least-squares (AGLS) method. The AGLS method combines the least-squares (LS) tests implemented by EPISNP1mpi [20,21] with the estimated breeding values from a routine genetic evaluation using the entire U.S. Holstein population. The statistical model was:

(1) $$y=\mu I+X_{g}g+Za+e=Xb+Za+e$$

where y is the column vector of phenotypic deviation after removing fixed nongenetic effects, such as heard-year-season (termed as ‘yield deviation’ for any trait) using a standard procedure for the CDCB/USDA genetic and genomic evaluation; μ is the common mean; I is the identity matrix; g is the column vector of SNP genotypic values; $X_{g}$ is the model matrix of g; $b=(\mu ,\;g^{\prime }{)}^{\prime }$, $X=(I,\;X_{g})$; a is the column vector of additive polygenic values; Z is the model matrix of a; and e is the column vector of random residuals. The first and second moments of Equation (1) are E(y)=Xb and $\mathrm{var}(y)=V={ZGZ}^{\prime }+R=\sigma_{a}^{2}{ZAZ}^{\prime }+\sigma_{e}^{2}I$, respectively, where $\sigma_{a}^{2}$ = additive variance, A = additive relationship matrix, and $\sigma_{e}^{2}$ = residual variance. The problem of estimating the **b** vector that includes SNP genotypic values in Equation (1) is the requirement of inverting **V** if the generalized least-squares (GLS) method is used or inverting the **A** matrix if the mixed model equations (MME) [22] are used. However, both V and A could not be inverted for our sample size. To avoid inverting these large matrices, the GWAS used the method of approximate GLS (AGLS), which replaces the polygenic additive values (a) with the best linear unbiased prediction based on pedigree relationships [4]. The AGLS method is based on the following results:

(2) $$\hat{b}={\left(X^{\prime }V^{-1}X\right)}^{-}X^{\prime }V^{-1}y$$

(3) $$\hat{b}={\left(X^{\prime }R^{-1}X\right)}^{-}(X^{\prime }R^{-1}y-X^{\prime }R^{-1}Z\hat{a})={\left(X^{\prime }X\right)}^{-}X^{\prime }(y-Z\hat{a})={\left(X^{\prime }X\right)}^{-}X^{\prime }y_{*}$$

where $y_{*}=y-Z\hat{a}$ and $\hat{a}$ is the best linear unbiased prediction (BLUP) of a. Equation (2) is the GLS solution, and Equation (3) is the MME solution of b. These two equations yield identical results, and $\hat{b}$ from either equation is termed the best linear unbiased estimator (BLUE) [22]. If $\hat{a}$ is known, the LS version of BLUE given by Equation (3) is computationally efficient relative to the GLS of Equation (2), requiring the V inverse, or the joint MME solutions of $\hat{b}$ and $\hat{a}$, requiring the A inverse. The AGLS method uses two approximations. The first approximation is to use $\tilde{a}$ from routine genetic evaluation as an approximation of $\hat{a}$ in Equation (3):

(4) $$\hat{b}={\left(X^{\prime }X\right)}^{-}X^{\prime }(y-Z\tilde{a})={\left(X^{\prime }X\right)}^{-}X^{\prime }y^{*}$$

where $y^{*}=y-Z\tilde{a}$, and $\tilde{a}$ is the column vector of 2(PTA) with PTA being the predicted transmission ability from the routine genetic evaluation. Equation (4) achieves the benefit of sample stratification correction from mixed models using pedigree relationships without the computing difficulty of inverting V or A. The second approximation of the AGLS approach is the *t*-test using the LS rather than the GLS formula of the t-statistic to avoid using the **V** inverse in the GLS formula. The significance tests for additive and dominance SNP effects used the *t*-tests of the additive and dominance contrasts of the estimated SNP genotypic values [20,23]. The t-statistic of the AGLS was calculated as:

(5) $$t_{j}=\frac{{|L}_{j}|}{\sqrt{{\mathrm{var}(L}_{j})}}=\frac{\left|s_{j}^{}\hat{g}\right|}{v\sqrt{s_{j}^{}{\left(X_{}^{\prime }X_{}\right)}_{\mathrm{gg}}^{-}s_{j}^{\prime }}},\;\;j=a,d$$

where $L_{j}$ is the additive or dominance contrast; $\sqrt{{\mathrm{var}(L}_{j})}$ is the standard deviation of the additive or dominance contrast; **s**_a_ represents the additive contrast coefficients ($P_{11}{/p}_{1}$, $0{.5P}_{12}{(p}_{2}-p_{1})/(p_{1}p_{2})$, −$P_{22}{/p}_{2}$); **s**_d_ represents the dominance contrast coefficients (−0.5, 1, −0.5); $v^{2}=\left(y-X\hat{b}{)}^{\prime }(y-X\hat{b}\right)/(n-k)$ is the estimated residual variance; $\hat{g}$ is the column vector of the AGLS estimates of the three SNP genotypic effects of $g_{11}$, $g_{12}$, and $g_{22}$ from Equation (4); ${\left(X_{}^{\prime }X_{}\right)}_{\mathrm{gg}}^{-}$ is the submatrix of ${\left(X_{}^{\prime }X_{}\right)}_{}^{-}$ corresponding to $\hat{g}$; $p_{1}$ is the frequency of $A_{1}$ allele; $p_{2}$ is the frequency of $A_{2}$ allele of the SNP; $P_{11}$ is the frequency of $A_{1}A_{1}$ genotype; $P_{12}$ is the frequency of $A_{1}A_{2}$ genotype; $P_{22}$ is the frequency of $A_{2}A_{2}$ genotype, n is the number of observations, and k is the rank of **X**. The formula of **s**_a_ defined above allows the Hardy–Weinberg disequilibrium [23] and simplifies to ($p_{1}$, $p_{2}-p_{1}$, −$p_{2}$) under the Hardy–Weinberg equilibrium.

Additive effects of each SNP were estimated using three measures, the average effect of gene substitution, allelic mean, and allelic effect of each allele based on quantitative genetics definitions [23,24]. The allelic mean ($\mu_{i}$), the population mean of all genotypic values of the SNP (μ), the allelic effect ($a_{i}$), and the average effect of gene substitution of the SNP (α) are:

(6) $$\mu_{1}=P_{11.1}g_{11}+0{.5P}_{12.1}g_{12}$$

(7) $$\mu_{2}=0{.5P}_{12.2}g_{12}{+P}_{22.2}g_{22}$$

(8) $$\mu =\sum_{i=1}^{2}p_{i}\mu_{i}$$

(9) $$a_{i}\;=\;\mu_{i}-\mu$$

(10) $$\alpha \;\;=\;L_{a}=s_{a}^{}\hat{g}\;=\;a_{1}-a_{2}=\;\;\mu_{1}-\mu_{2}$$

where $P_{11.1}=P_{11}{/p}_{1}$, $P_{12.1}=P_{12}{/p}_{1}$, $P_{12.2}=P_{12}{/p}_{2}$, and $P_{22.2}=P_{22}{/p}_{2}$. The additive effect measured by the average effect of gene substitution of Equation (10) is the difference between the two allelic means or effects of the same SNP, and it is the fundamental measure for detecting SNP additive effects, as shown by the t-statistic of Equation (5). The allelic effect defined by Equation (9) provide an understanding of the effect size and direction of each allele. However, the allelic effect of Equation (9) is not comparable across SNPs because the allelic effect is affected by the genotypic mean of the SNP defined by Equation (8). To compare allelic effects across SNPs, we replace the SNP genotypic mean (μ) in Equation (9) with the average of all SNP genotypic means ($\mu_{\mathrm{all}}$):

(11) $$a_{i}\;=\;\mu_{i}-\mu_{\mathrm{all}}$$

The dominance effect of each SNP was estimated as the dominance contrast $\hat{g}$ from Equation (4), i.e.,

(12) $$\delta =L_{d}\;=\;d_{12}-{(d}_{11}{+d}_{22})/2\;\;=\;\;g_{12}-{(g}_{11}+g_{22})/2$$

where $g_{\mathrm{ij}}$ represents the AGLS estimates of SNP genotypic values from Equation (4) (i, j = 1, 2) and $d_{\mathrm{ij}}$ is the dominance value (dominance deviation) of the $A_{i}A_{j}$ SNP genotype

(13) $$d_{\mathrm{ij}}=g_{\mathrm{ij}}\;-\mu -a_{i}-a_{j}$$

In this study, overdominance refers to the fact that the dominance value of the heterozygous genotype is more extreme than that of either homozygous genotype, i.e., $d_{12}$ > $d_{11}$ and $d_{12}$ > $d_{22}$ for positive overdominance effects, or $d_{12}$ < $d_{11}$ and $d_{12}$ < $d_{22}$ for negative overdominance effects. The dominance effects to be reported were all positive overdominance effects. For 75,524 SNPs with additive and dominance effects, the threshold *p*-value for declaring significant *t*-tests for the Bonferroni correction with 0.05 genome-wide false positives was 10^−8^, or log_10_(1/*p*) = 8. All figures for the GWAS results were produced using SNPEVG2 in the SNPEVG package [25]. 

## 4. Conclusions

This large sample GWAS identified significant additive effects in three chromosome regions and implicated two reproductive hormone genes affecting AFC. A small number of significant positive overdominance effects were also identified. The results provided new evidence and understanding of the genetic variants and chromosome regions affecting AFC in U.S. Holstein cows.

## Figures and Tables

**Table 1:** Top 20 significant additive effects for AFC.

SNP	Chr	Position (bp)	Candidate Gene	Effect (α, −Days)	al+	ae+ (−Days)	f_al+	al−	ae− (−Days)	f_al−	log_10_(1/*p*)
rs110401500	19	31,252,963	*ARHGAP44*	−1.02	2	0.730	0.287	1	−0.294	0.713	37.30
rs41257332	19	33,443,229	*TTC19*	−0.97	2	0.616	0.366	1	−0.355	0.634	36.45
rs111004845	19	27,355,811	*SHBG* (9664 bp u) ^a^	−0.97	2	0.648	0.332	1	−0.323	0.668	36.06
rs135712994	19	33,421,057	*NCOR1*	−0.89	2	0.359	0.597	1	−0.531	0.403	33.14
rs110761858	19	33,358,794	*NCOR1*	−0.87	2	0.354	0.592	1	−0.514	0.408	31.87
rs41621822	19	31,902,307	*LOC112442639*	−0.90	2	0.594	0.339	1	−0.304	0.661	31.39
rs133729181	19	32,106,657	*COX10*	0.86	1	0.519	0.394	2	−0.337	0.606	30.40
rs134054295	23	32,599,962	*LOC537017*	−1.02	2	0.808	0.211	1	−0.217	0.789	30.30
rs136368496	23	28,526,405	*LOC101905956*	−0.87	2	0.555	0.364	1	−0.318	0.636	30.04
rs110845473	19	27,484,633	*DNAH2*	−0.98	2	0.753	0.234	1	−0.230	0.766	29.89
rs41904669	19	27,073,319	*TNK1*	0.97	1	0.738	0.239	2	−0.232	0.761	29.43
rs109836072	15	7,475,196	*TRPC6* (3151 bp d) ^a^	−1.04	2	0.818	0.212	1	−0.220	0.788	29.38
rs137457305	23	26,926,436	*C23H6orf10*	0.98	1	0.746	0.236	2	−0.231	0.764	29.28
rs41904556	19	27,316,118	*MPDU1*	0.96	1	0.732	0.240	2	−0.231	0.760	29.03
rs42688274	19	29,273,714	*GAST* (22,315 bp d)	−0.97	2	0.740	0.235	1	−0.227	0.765	29.03
rs29010491	23	30,176,828	*ENSBTAG00000051232* (21,566 bp u) ^a^	0.81	1	0.442	0.453	2	−0.366	0.547	28.03
rs137317833	23	29,958,908	blank	−0.81	2	0.455	0.441	1	−0.358	0.559	27.93
rs136764006	15	7,861,416	*PGR*	0.89	1	0.620	0.303	2	−0.270	0.697	27.63
rs110654893	23	30,013,004	*ZNF311* (5017 bp u) ^a^	−0.80	2	0.438	0.453	1	−0.363	0.547	27.53
rs109681200	23	30,377,501	*ZSCAN31*	−0.83	2	0.538	0.354	1	−0.294	0.646	27.39

^a^ ‘u’ indicates the SNP is upstream of the gene, and ‘d’ indicates the SNP is downstream of the gene. ‘effect’ is the additive effect of the SNP as the difference between allelic effects of ‘allele 1’ and ‘allele 2’ (Equation (10)). ‘ae+’ is the allelic effect of the positive allele (Equation (11)). ‘ae−’ is the allelic effect of the negative allele (Equation (11)). ‘f_al+’ is the frequency of the positive allele. ‘f_al−’ is the frequency of the negative allele.

**Table 2:** Top 10 significant dominance effects for AFC.

SNP	Chr	Position	Candidate Gene	Effect (δ, −Days)	DR	d_DR (−Days)	f_DR	DD	d_DD (−Days)	f_DD	RR	d_RR (−Days)	f_RR	f_R	log_10_(1/*p*)
rs109438971	5	26,964,045	*EIF4B* (14,636 bp d)	5.53	12	0.62	0.152	22	−0.06	0.843	11	−9.76	0.005	0.081	45.08
rs110558219	5	26,715,326	*AAAS*	5.51	12	0.62	0.152	11	−0.06	0.843	22	−9.72	0.005	0.081	44.89
rs43768813	6	101,887,271	*AFF1*	4.87	12	0.61	0.131	22	−0.05	0.864	11	−8.48	0.005	0.07	33.36
rs42739334	6	102,065,812	*KLHL8*	4.68	12	0.60	0.136	11	−0.05	0.859	22	−8.11	0.005	0.073	33.04
rs109675908	5	26,499,453	*ATF7*	3.88	12	0.54	0.170	22	−0.05	0.823	11	−6.63	0.007	0.092	30.77
rs43480825	6	101,994,654	*AFF1*	4.57	12	0.57	0.135	11	−0.04	0.860	22	−7.95	0.005	0.072	30.56
rs109933750	6	102,164,971	*U6* (16,972 bp d)	4.50	12	0.56	0.134	22	−0.04	0.861	11	−7.83	0.005	0.072	29.18
rs135494774	5	25,556,149	*NCKAP1L*	3.46	12	0.53	0.180	11	−0.06	0.811	22	−5.80	0.008	0.099	27.34
rs134764130	5	26,385,947	*ATP5MC2* (7720 bp d)	3.14	12	0.51	0.192	22	−0.06	0.798	11	−5.18	0.010	0.106	26.13
rs41603412	5	33,076,713	*PCED1B*	3.17	12	0.50	0.185	22	−0.06	0.806	11	−5.28	0.009	0.101	25.56

‘d’ indicates the SNP is downstream of the gene. ‘effect’ is the dominance effect of the SNP as the difference between the heterozygous dominance value and the average of the two homozygous dominance values (Equation (12)). ‘DR’ is the heterozygous genotype with one dominant allele and one recessive allele. ‘d_DR’ is the dominance value of the heterozygous genotype with one dominant allele (D) and one recessive allele (R) (Equation (13)). ‘DD’ is the homozygous genotype with two dominant alleles. ‘d_DD’ is the dominance value of the homozygous genotype with two dominant alleles (DD) (Equation (13)). ‘RR’ is the homozygous genotype with two recessive alleles. ‘d_RR’ is the dominance value of the homozygous genotype with two recessive alleles (RR) (Equation (13)). ‘f_DR’ is the frequency of the heterozygous genotype. ‘f_DD’ is the frequency of the homozygous genotype of the dominant allele. ‘f_RR’ is the frequency of the homozygous genotype of the recessive allele. ‘f_R’ is the frequency of the recessive allele.

**Table 3:** Negative impact of recessive genotypes of seven SNPs on AFC and three yield traits.

SNP	Formula of Negative Impact ^a^	AFC (Days)	Milk Yield (kg)	Fat Yield (kg)	Protein Yield (kg)
rs109675908	$y_{11}-{(y}_{12}{+y}_{22})/2$ ^b^	7.69	−470.06	−20.14	−14.22
rs110558219	$y_{22}-{(y}_{11}{+y}_{12})/2$	10.75	−646.33	−26.03	−19.27
rs109438971	$y_{11}-{(y}_{12}{+y}_{22})/2$	10.88	−640.94	−26.03	−19.11
rs43768813	$y_{11}-{(y}_{12}{+y}_{22})/2$	12.50	−169.35	−9.05	−5.73
rs43480825	$y_{22}-{(y}_{11}{+y}_{12})/2$	12.00	−189.78	−9.88	−6.40
rs42739334	$y_{22}-{(y}_{11}{+y}_{12})/2$	12.83	−240.84	−10.50	−7.41
rs109933750	$y_{11}-{(y}_{12}{+y}_{22})/2$	11.76	−201.23	−10.22	−6.74

^a^ Negative impact of a recessive genotype is defined as the difference between the average of the phenotypic values of cows carrying the recessive genotype and the average of the phenotypic values of cows carrying the heterozygous genotypes and the homozygous dominant genotypes. ^b^ $y_{\mathrm{ij}}$ is the average of the phenotypic values of cows with SNP genotype ij (i,j = 1,2), and $y_{\mathrm{ij}}$ values are given in Table S4. The ‘11’ genotypes of four SNPs were the recessive genotypes, and the ‘22’ genotypes of the remaining three SNPs were the recessive genotypes.

## Data Availability

The original genotype data are owned by third parties and maintained by the Council on Dairy Cattle Breeding (CDCB). A request to CDCB is necessary for getting data access on research, which may be sent to: João Dürr, CDCB Chief Executive Officer (joao.durr@cdcb.us). All other relevant data are available in the manuscript and Supplementary Materials.

## Supplementary Materials

The following are available online: https://www.mdpi.com/article/10.3390/ijms24087109/s1.
