# Supplementary material for: Genome-Wide Association Study of Age at First Calving in U.S. Holstein Cows
Source: Int J Mol Sci. 2023 Apr 12;24(8):7109. doi: 10.3390/ijms24087109 (PMC10138929; doi:10.3390/ijms24087109)
Supplement: Supplementary file 1 [file ijms-24-07109-s001.zip › Supplementary_material.pdf]

Table S1. Basic statistics of AFC phenotypic residuals after removing fixed nongenetic effects from the 12/2021 U.S. Holstein genetic evaluation.

Trait	Counts	Mean (-days)	Min (-days)	Max (-days)	SD (days)
AFC	813114	12.02	-405.17	363.12	46.15

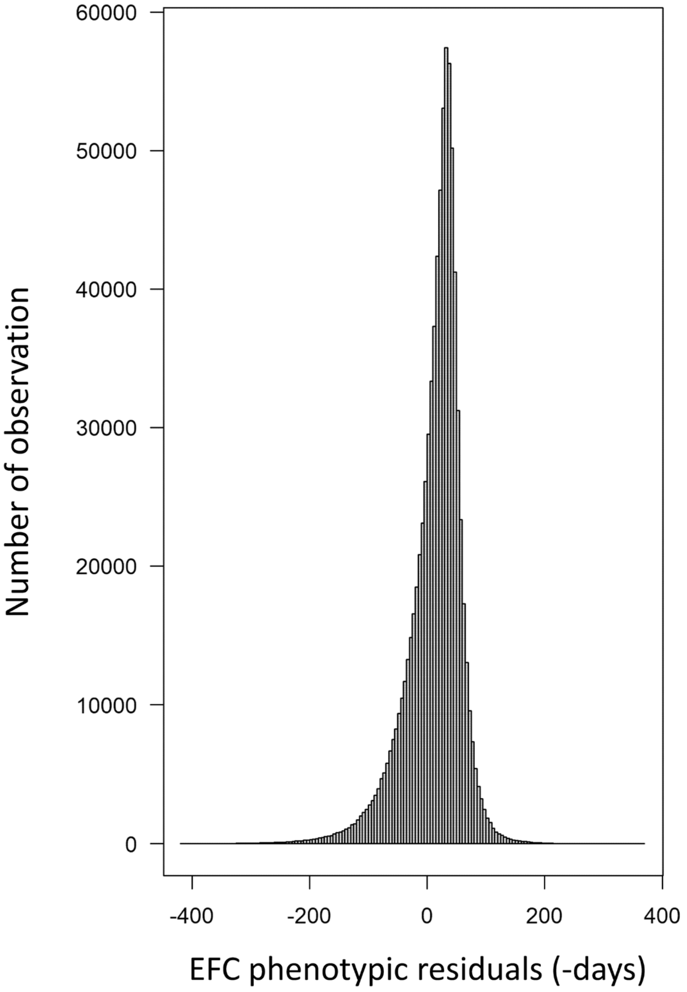

Figure S1. Distribution of AFC phenotypic residuals after removing fixed nongenetic effects from the 12/2021 U.S. Holstein genetic evaluation.

Table S2. Significant additive effects. (Excel)

Table S3. Significant dominance effects. (Excel)

Table S4. Average phenotypic values and negative impact of recessive genotypes of seven SNPs. (Excel)

Table S5. Gene Ontology biological processes of candidate genes with additive effects. (Excel)

Table S6. Gene Ontology biological processes of candidate genes with dominance effects. (Excel)
